# Supplementary material for: Learning curve and surgical outcome of robotic assisted colorectal surgery with ERAS program
Source: Sci Rep. 2022 Nov 29;12:20566. doi: 10.1038/s41598-022-24665-w (PMC9709162; doi:10.1038/s41598-022-24665-w)
Supplement: Supplementary file 2 — Supplementary Information 2. [file 41598_2022_24665_MOESM2_ESM.zip › RAL Raw data-Ñ[▒K/078/OP N_ñw╝╨░O▒Kñσ.pdf]

W95 / W95

臺中榮民總醫院
手術室護理記錄

[redacted]

基本資料

- 室別： 開刀房-17室，手術科別： CRS，手術類別： 常規，日期： 2021/04/23
- 進入時間： 1620 ； 麻醉開始時間： 1635 ； Time Out時間： 1830 ； 劃刀時間： 1840 ； 手術結束時間： 0015 送出時間： 0055 ；
- 麻醉方式： GE
  診斷：Constipation – functional, change
  手術名稱：Robot Assisted Colectomy(L)
  手術局部用藥：NIL.
  術前預防性抗生素：
  - 抗生素名稱一： Veterin 1GM IVD由麻醉護理師給予
  - 時間： 1820
  - 上次簽章: 陳佳琪

  ，追加預防性抗生素：
  - 抗生素名稱一： Veterin 1GM IVD由麻醉科護理人員給予
  - 時間： 2220
  - 上次簽章: 陳佳琪
- 手術醫師： 蔣鋒帆，黃佀霖，林陳彥，陳勇先
- 麻醉醫師： VS 蔡佩馨
-
  - 刷手護士A ： 邱玟琇，時間： 1620，刷手護士B ： 陳佳琪，時間： 1620，接替刷手一： 張秀敏，時間： 1800，接替刷手二： 高子貽，時間： 2230
  - 流動護士A ： 張秀敏，時間： 1620，接替流動一： 陳佳琪，時間： 1730，接替流動二： 林愛甯，時間： 2200
- 更多接替及流動護士： 接替刷手三： 章佑禎，時間： 2300，接替流動三： 許育綾，時間： 2300

皮膚狀況

A-V Shunt： 無
術前： 完整
記錄者： 上次簽章: 張秀敏
術後： 完整
記錄者： 許育綾

手術準備

手術臥式 單一手術臥式

-
  - 手術臥式： 截石臥式
  - 使用附件： 溫毯 38 ℃，頭圈，腳架，側身架，其它： 海綿*4、頭架.
  - 皮膚準備： 10%Tr.Betadine，10%Aq Betadine，Hibiscrub
  - 電燒： 使用電燒: 電極片位置： 右大腿，雙極電燒，記錄者： 上次簽章: 張秀敏

止血帶T： 無

植入物

無

標本送檢

- 細菌培養 無
- 細胞學 無
- 冰凍切片 無
- 病理標本
  組織名稱一：COLON
- 截肢 無

標本交付 標本： 1 袋 ， 標本： 0 盒 ， 標本： 0 瓶 ， 細胞學： 0 管 ， Culture Tube ： 0 管 ， 被交付醫師： R 莊雅棋 ，

- 記錄者： 許育綾
- 時間： 0050

傷口術後敷料

傷口敷料：　　　　　　傷口敷料： 無

- 請於圖上標示部位：

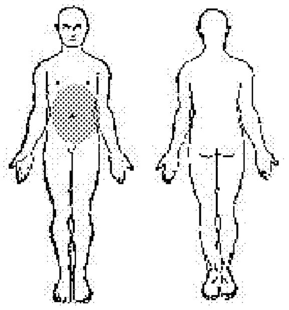

腹部.

- 傷口敷料： gauze

導管

導管	天數	導管資訊	插入日期	插入時間	更換日期	拔除日期	拔除時間	拔除原因	預計拔除/更換	預計拔除/更換日期
Foley(1)	2	號碼:16 Fr.; 部位:膀胱;	2021/04/23	1800						
Epidural PCA(1)	2	部位:硬脊膜;	2021/04/23	1800						
CVC(1)	2	號碼:8 Fr.; 深度:15cm; 部位:jugular (右); 象限:RU;	2021/04/23	1800						
A-line (1)	2	號碼:20 Ga.; 部位:右手;	2021/04/23	1800						

用物計數

- 手術中出血量： minimal c.c.
- 手術中尿量： 750 c.c.
-
    - 其它： 無

用物計數： 多切口同時縫合

- 1.器械： 正確 ，2.縫針： 正確 ，3.刀片： 正確 ，4.紗布： 正確 ，5.腹部墊： 未使用 ，6.紗球： 未使用 ，7.PEANUTS ： 未使用 ，8.紗條： 未使用 ，9.絲帶： 未使用 ，10.針頭： 未使用 ，11.Vessel loop： 未使用 ，12.Boots： 未使用 ，13.Jelco： 未使用 ，14.棉條： 未使用 ，
    - 第一次：
        - 刷手護士 ： 高子貽
        - 流動護士： 上次簽章: 林愛宵
        - 時間： 2230

  ,
    - 第二次：
        - 刷手護士 ： 章佑禎
        - 流動護士： 許育綾
        - 時間： 2315

  ,
    - 第三次：
        - 刷手護士 ： 章佑禎
        - 流動護士： 許育綾
        - 時間： 2345

備註

備註： davinci XI scope 30度2號.

- 記錄者： 上次簽章: 張秀敏
- 時間： 1700

填寫護理人員 許育綾

正式摘要

W95 / W95

doctorRank:B
